# *In vivo* nose-to-brain delivery of the hydrophilic antiviral ribavirin by microparticle agglomerates

**DOI:** 10.1080/10717544.2018.1428242

**Published:** 2018-01-30

**Authors:** Alessandro Giuliani, Anna Giulia Balducci, Elisa Zironi, Gaia Colombo, Fabrizio Bortolotti, Luca Lorenzini, Viola Galligioni, Giampiero Pagliuca, Alessandra Scagliarini, Laura Calzà, Fabio Sonvico

**Affiliations:** a Department of Veterinary Medical Science, Alma Mater Studiorum – University of Bologna, Ozzano, Italy;; b Department of Food and Drug, University of Parma, Parma, Italy;; c Interdepartmental Center for Health Products – Biopharmanet TEC, University of Parma, Parma, Italy;; d Department of Life Sciences and Biotechnology, University of Ferrara, Ferrara, Italy;; e IRET Foundation, Ozzano, (BO), Italy;; f Department of Pharmacy and Biotechnology, Ozzano, Italy

**Keywords:** Ribavirin, microparticles, agglomerates, nasal administration, brain delivery

## Abstract

Nasal administration has been proposed as a potential approach for the delivery of drugs to the central nervous system. Ribavirin (RBV), an antiviral drug potentially useful to treat viral infections both in humans and animals, has been previously demonstrated to attain several brain compartments after nasal administration. Here, a powder formulation in the form of agglomerates comprising micronized RBV and spray-dried microparticles containing excipients with potential absorption enhancing properties, i.e. mannitol, chitosan, and α-cyclodextrin, was developed for nasal insufflation. The agglomerates were characterized for particle size, agglomeration yield, and *ex vivo* RBV permeation across rabbit nasal mucosa as well as delivery from an animal dry powder insufflator device. Interestingly, permeation enhancers such as chitosan and mannitol showed a lower amount of RBV permeating across the excised nasal tissue, whereas α-cyclodextrin proved to outperform the other formulations and to match the highly soluble micronized RBV powder taken as a reference. *In vivo* nasal administration to rats of the agglomerates containing α-cyclodextrin showed an overall higher accumulation of RBV in all the brain compartments analyzed as compared with the micronized RBV administered as such without excipient microparticles. Hence, powder agglomerates are a valuable approach to obtain a nasal formulation potentially attaining nose-to-brain delivery of drugs with minimal processing of the APIs and improvement of the technological and biopharmaceutical properties of micronized API and excipients, as they combine optimal flow properties for handling and dosing, suitable particle size for nasal deposition, high surface area for drug dissolution, and penetration enhancing properties from excipients such as cyclodextrins.

## Introduction

Viral diseases are considered a major global threat to human and veterinary public health (Howard & Fletcher, 2012). As emerging viral infections, they frequently originate from an animal host and many involve the central nervous system (CNS) causing encephalitis, meningitis and consequent acute flaccid paralysis/poliomyelitis, as in the case of West Nile Virus and others (Tyler, 2009). Also measles was recently added to the NIAID's list of emerging virus infections and may cause CNS complications soon after infection (Garg, 2008) or few years later, as a result of viral persistence that leads to subacute sclerosing panencephalitis (Mahajan et al., 2014). The treatment and control of viral infections in the CNS is a therapeutic challenge because of the blood–brain barrier (BBB) and the blood–cerebrospinal fluid barrier. The extensive tight junctions between the endothelial cells of brain capillaries, the lack of paracellular transport, and limited pinocytosis, together with active efflux transporters, result in low cerebrospinal fluid-plasma ratios for most of the antiviral drugs (Wong et al., 2012; Comfort et al., 2015).

The nasal route has been extensively studied for the administration of drugs directly to the CNS (Landis et al., 2012). In fact, this route exploits the olfactory region and the trigeminal nerve pathway to enable drugs’ entry into the CNS bypassing the BBB (Hanson & Frey, 2008). This delivery approach was explored for the administration of ribavirin (RBV), in view of an innovative treatment of the encephalitis associated with canine distemper virus, a major veterinary infection that could serve as a proof of concept of the approach. This virus belongs to the *Morbillivirus* genus as the measles virus and primarily infects dogs (Elia et al., 2008; Dal Pozzo et al., 2010). RBV is a synthetic guanosine antiviral analog. It has a broad-spectrum antiviral activity, being clinically effective against several viruses and successfully tested *in vitro* against several RNA and DNA virus infections (Beaucourt & Vignuzzi, 2014). The drug is currently marketed in oral dosage forms; however, it has been clinically administered as pulmonary aerosol in the treatment of respiratory syncytial virus (Li et al., 2012) and for the therapy of measles pneumonia (Safdar et al., 2009) as well as intravenously and intraventricularly for subacute sclerosing panencephalitis (Tomoda et al., 2003; Garg, 2008). However, the latter two approaches are plagued by adverse effects and exploit a highly invasive, risky, and infection-prone administration route, remarkably fostering the development of an alternative delivery route such as nasal administration. Our group showed that measurable brain concentrations of RBV can be obtained in rats after intranasal administration of a RBV aqueous solution. In particular, it was evidenced that 20 min after nasal administration, RBV was highly concentrated in the olfactory bulb and in other brain structures after nasal administration (Colombo et al., 2011).

Currently, despite liquid dosage forms prevail in nasal drug delivery, a powder formulation would be preferable from a chemical and biopharmaceutical perspective. In fact, liquids are plagued by limited residence time in the nasal cavity, chemical and microbiological stability issues, and limit the deliverable dose due to the drug solubility and the maximal concentration attainable in aqueous vehicles (Buttini et al., 2012). In this regard, the superiority of nasal powders has been demonstrated *in vitro* (Colombo et al., 2016; Pozzoli et al., 2017), in animal studies (Balducci et al., 2013b), and in clinical trials (Fransén et al., 2009).

In light of this, the aim of this work was to formulate RBV as a nasal powder with appropriate physico-chemical properties for nasal delivery and containing a functional excipient that could act as penetration enhancer to improve the nose-to-brain delivery of the antiviral drug.

Micronized RBV appears as a promising starting material, with rapid dissolution assured by the high surface area of the micron-sized crystals. However, its technological properties are poor. In particular, bad powder flow and high cohesiveness, typical of micronized powders, may hinder handling, dosing, and powder aerosolization for intranasal deposition.

In order to improve the technological properties of the micronized drug powder, it was previously demonstrated that lecithin-containing spray-dried microparticles could be used to form agglomerates by simple mechanical processes (Sacchetti et al., 2002). These soft agglomerates were of a size sufficiently large to allow for precise handling and dosing, but weak enough to break upon insufflation and rapidly dissolve in the presence of aqueous secretions (Russo et al., 2004).

Here, agglomerates were prepared by mixing the RBV raw material with excipient microparticles obtained by spray drying. These excipient microparticles were composed of lecithin associated with a hydrophilic component among mannitol, chitosan, or α-cyclodextrin. Mannitol has been demonstrated to alter mucosal permeability by osmotic effect (Hirsh, 2002; Farkas et al., 2005). Chitosan is a cationic polysaccharide with bioadhesive and permeation enhancing properties through transient opening of tight junctions (Caramella et al., 2010). Cyclodextrins are well known for their nasal bioavailability enhancement, by various mechanisms including deprival of cholesterol from the mucosal membrane and improved paracellular drug transport (Rassu et al., 2015; Kulkarni & Avachat, 2017). Hence, RBV microcrystals were agglomerated with microparticulate excipients with potential permeation enhancing properties.

RBV, excipient microparticles, and agglomerates were characterized *in vitro* with respect to size distribution, morphology, and technological properties. *Ex vivo* transport experiments across rabbit nasal mucosa were conducted in order to compare RBV agglomerates with the RBV aqueous solution and RBV raw material, both used as reference. Finally, the agglomerate formulation that showed the best performance *in vitro* was administered intranasally to rats.

## Materials and methods

### Materials

Ribavirin raw material (batch #001-RIB-0908) was kindly donated by Euticals S.p.A. (Lodi, MI, Italy). The micronized powder was demonstrated to be exclusively the crystalline polymorph R-I by X-ray diffraction (data not shown) and DSC (*T*_m_ =180 °C), probably as a result of the milling process as reported by Vasa (Vasa & Wildfong, 2017). RBV analytical standard was purchased from Sigma Chemical Company (St. Louis, MO). The internal standard (IS) ^13^C_5_RBV was obtained from Campro Scientific GmbH (Berlin, Germany). Mannitol was a kind gift of Lisapharma S.p.A. (Erba, Italy). Chitosan (ChitoClear®, batch TM1874) was supplied by Primex (Siglufjordur, Iceland). The oligosaccharide α-cyclodextrin (α–CD, A.C.E.F. S.p.A., Fiorenzuola d’Arda, Italy). Lecithin (LIPOID S45) was obtained from Lipoid GmBH (Ludwigshafen, Germany). Degassed ultrapure water (Purelab Flex, ELGA-Veolia LabWater, Zoppola, Italy) was used in all experiments. All other reagents and solvents were of analytical grade.

### Methods

#### RBV chromatographic analysis

##### HPLC method for *in vitro* and *ex vivo* experiments

The RBV concentration in samples from *in vitro* experiments was quantified by a reverse phase-HPLC method previously described (Colombo et al., 2011). Briefly, RBV was quantified using a Synergi Polar-RP 4 µm, 150 mm × 4.6 mm (Phenomenex, Castelmaggiore, Italy). Separation was obtained by isocratic elution carried out with a 20 mM (NH_4_)_2_HPO_4_ solution in water (pH 7.5) as mobile phase. The analyses were carried out at room temperature, flow rate 0.8 ml/min and detection wavelength 225 nm. The injection volume was 20 μl. In these conditions, the retention time of ribavirin was around 3.7 min. The method was linear (*R*^2^ = 0.999) in the concentration range between 1.28 and 410 µg/ml, RSD (*n* = 6) was 0.34%, limit of quantitation (LOQ) and limit of detection (LOD) were 0.056 and 0.017 µg/ml, respectively, and theoretical plates were 4553 ± 339.

##### LC-MS/MS method for *in vivo* experiments

For the *in vivo* studies, RBV concentration in brain and blood extracts was determined according to a method already published (Zironi et al. 2011). Briefly, after the addition of ^13^C_5_RBV (IS), RBV was extracted from samples with ammonium acetate buffer. The analyses were conducted on an Alliance 2695 Separations Module coupled with triple quadrupole mass spectrometer Quattro Premiere XE, (Waters Corporation, Milford, MA). Chromatographic separation was performed on a Waters Atlantis T3 column (3 μm, 2.1 mm ×150 mm). All the analyses were conducted in positive electrospray ionization mode (ESI+) using selected reaction monitoring (SRM); the transitions monitored were RBV *m*/*z* 245→113, ^13^C_5_RBV *m*/*z* 250→113. The LOQ was 5 ng/g and the linearity (*R*^2^>0.99) was demonstrated over a concentration range of 5–1000 ng/g.

##### Spray-dried excipient microparticle manufacturing

The excipient microparticles were constituted by two components blended at mass ratio 15:85. The first component was always soybean lecithin, that confers adhesive properties, whereas the second one was a hydrophilic excipient with functional properties, namely mannitol, chitosan, or α–cyclodextrin (α–CD). The corresponding excipient microparticles were coded M1, M2, and M3.

The excipient microparticles were manufactured by means of a B-191 Mini Spray Dryer (Büchi, Flawil, Switzerland) under the following operating conditions: airflow 600 l/h and aspiration 35 m^3^/h. The feeding solution was prepared by separately solubilizing lecithin in ethanol and mannitol or α–CD in ultrapure water (Purelab Flex, ELGA-Veolia LabWater, Windsor Court, UK). Chitosan was dissolved in a 1% w/w lactic acid solution (pH 2.4). The lecithin solution was then added to the aqueous solution at volume ratio 1:10 under magnetic stirring. The total solid concentration in the feed solution was 2% w/v. The liquid feed was sprayed at 4 ml/min.

The inlet and the outlet temperatures of the air for the drying process were 130 and 68 °C for M1, 120 and 52 °C for M2 and 105 and 54 °C for M3 microparticles preparation.

Percentage process yields were calculated as the ratio between the weight of the powder recovered in the collector at the end of the process and the amounts of materials weighed for the preparation of the feeding solution.

##### Microparticle agglomeration

Before the agglomeration process, the crystalline powder of micronized ribavirin (µRBV) was blended with the spray-dried excipient microparticles at 1:1 mass ratio. One gram of each powder was weighed inside a pillbox of 30 ml capacity with two glass spheres (7.83 mm in diameter) and then mixed in a Turbula^®^ blender (WAB, Muttenz, Switzerland) for 10 min. Then, as the mixture partially adhered to the container’s wall, it was gently scraped off with a spatula and the powder further mixed for 10 min. Blend homogeneity with respect to drug content was assessed by HPLC by dissolving 10 mg of the blend in mobile phase.

For agglomeration, 2 g of the blend were placed on top of a 2-sieve nest (10 cm diameter sieves, Endecotts Ltd, London, UK; nominal aperture 500 and 300 µm, respectively) on a laboratory sieve shaker (Vibratory Sieve Shaker, Retsch^®^, Haan, Germany) (Russo et al., 2006; Balducci et al., 2013a). The system was vibrated for 5 min at an amplitude of 4/5. The agglomerates retained on the 300 µm sieve were collected, weighed to calculate the percentage agglomeration yield (ratio between the weight of the agglomerates on the sieve and the amounts of materials processed), and stored in tightly closed containers for further studies. Reprocessing of non-agglomerated powder was repeated five times. The drug loading of the agglomerates was evaluated by HPLC by directly dissolving 10 mg of the agglomerates in mobile phase.

#### Microparticle and agglomerate characterization

##### Scanning electron microscopy analysis

The morphology of RBV raw material, spray-dried excipient microparticles and agglomerates was examined by Field Emission – Scanning Electron Microscopy (FE-SEM), using a Zeiss SUPRA 40 microscope (Zeiss, Oberkochen, Germany). The powder samples were fixed onto high-purity aluminum pin stubs using double-sided tape, with the exception of the agglomerates which were dipped in silver glue (Conductive Silver Paint, Agar Scientific, Stansted, UK), as it was not possible to stick them onto the bioadhesive tape without breaking them. This paint formed a thin highly conductive film on the agglomerate’s surface. In all cases, the magnifications selected ranged between 250× and 5000× to appreciate both the whole particle and its surface detail.

##### Water content analysis

Residual water content in RBV raw material and in excipients microparticles was determined by Karl-Fisher titration (TitroMatic KF 1 S, Crison, Barcelona, Spain). Samples were analyzed in triplicate using around 100 mg of powder and using sodium tartrate tetrahydrate as standard.

##### Particle and agglomerate size distribution and flowability

Size distribution analysis of µRBV raw material and spray-dried excipient microparticles was carried out by a wet method using a Spraytec^®^ laser diffractometer (Malvern Instruments, Malvern, UK). Approximately, 10 mg of excipient microparticles were suspended in acetone and measured using a particle and dispersant refraction index of 1.000 and 1.3588, respectively. The analysis was conducted in triplicate for each batch.

The agglomerates’ size distribution was determined by optical microscopy (Citoval 2, Alessandrini, Modena, Italy) equipped with a digital camera (CCD-1, JVC, Tokyo, Japan). For the observation, agglomerates were gently spread under the objective in order to obtain an evenly spaced distribution. Images of the agglomerates were acquired and analyzed using the open source image processing package FiJi (Schindelin et al., 2012) to measure the projected area diameter of at least 800 agglomerates per batch. Finally, the cumulative percentile of the powders in the range between 300 µm and 1000 µm in a frequency distribution with 50 µm intervals was calculated using the Statplus software (AlaystSoft, Walnut, CA).

Powder flowability was evaluated by measuring the apparent volumes of micronized RBV and AM3 agglomerates according to the relevant Ph. Eur. chapter (2.9.36. Powder Flow) (SVM 122, Erweka, Heusenstamm, Germany). The measured bulk and tapped volume values were used to calculate the powder Compressibility (or Carr) Index (CI) according to the following equation: Measurements were done in triplicate.

(1) $$\text{C}\text{I}=\frac{{\text{V}}_{\text{B}\text{u}\text{l}\text{k}}-{\text{V}}_{\text{T}\text{a}\text{p}\text{p}\text{e}\text{d}}}{{\text{V}}_{\text{B}\text{u}\text{l}\text{k}}}\cdot 100$$

The calculated CI value is a parameter characteristic of powder flow properties with low values corresponding to a powder with excellent flow and high values to a powder with poor flow properties.

##### *Ex vivo* transport experiments across rabbit nasal mucosa

The *ex vivo* RBV transport from the agglomerates was investigated using vertical Franz-type diffusion cells (0.58 cm^2^ area) and rabbit nasal mucosa as barrier according to Bortolotti and colleagues (Bortolotti et al., 2009). Briefly, on the day of the experiment rabbit heads were collected from a local slaughterhouse (Pola, Finale Emilia, Italy) to dissect the epithelium specimens from the nasal septum. The relatively small size of rabbit nasal cavity does not allow to separate the olfactory from the respiratory region. The excised tissue was immediately inserted between the cell’s donor and receptor compartments with the mucosal side facing the donor compartment. The two compartments were tightly clamped. The receptor compartment was filled with phosphate buffered saline (PBS) pH 7.4 (4 ml) and maintained under stirring at 37 °C. Before adding the powder formulation, 200 µl of PBS was added to the donor to wet the mucosa. An amount of agglomerates equivalent to 5 mg of ribavirin or 0.5 ml of a ribavirin solution in water (0.1 mg/ml) was loaded into the donor compartment.

At predetermined time points, 0.4 ml of receptor solution was withdrawn and the receptor compartment refilled with an equivalent volume of fresh PBS. At the end of the experiment, the residual formulation on the membrane was quantitatively recovered by rinsing the donor compartment with PBS. All samples were analyzed by HPLC. The experiments were conducted in triplicate for each formulation.

##### *Ex vivo* powder insufflation with a dry powder insufflation device for small animals

A preliminary assessment of powder behavior during insufflation was carried out using a colored drug-free powder. In particular, the area of powder deposition on the nasal mucosa of a rat was investigated. The M3 spray-dried excipient microparticles were mixed with a methylene blue powder (85:15). The PennCentury^TM^ Dry Powder Insufflator™ – Model DP-4 (PennCentury Inc, Wyndmoor, PA) was used, which is designed to produce a puff of fine powder. The device was loaded with 3 mg of this blend and the powder insufflated into the nostril using a rat head. The head had been kept in the refrigerator for future use, deriving from an animal previously sacrificed for other purposes.

#### Intranasal administration of RBV

##### Animal care

Male pathogen-free Sprague–Dawley rats (Harlan^®^, Udine, Italy) weighing 125–150 g were used in this study. Animals were housed in standard conditions at 22 °C with 12-h light/dark cycle and received a standard pellet diet (4RF21, Mucedola, Settimo Milanese, Italy) with water available *ad libitum*. All animal experiments described here were carried out according to the European Community Council Directive of November 24th, 1986 (86/609/EEC) and approved by intramural committee and the Ministero della Salute (prot. 34613-X/10) in compliance with the guidelines published in Guide for the Care and Use of Laboratory Animals by the Italian National Research Council.

##### *In vivo* RBV agglomerate administration

The conditions for intranasal administration (including time-point for sacrifice) and CNS dissection for RBV quantification were described and optimized in the previous work by Colombo et al. (2011). All *in vivo* treatments were performed in anesthetized rats. Surgical anesthesia was induced by intraperitoneal injection of ketamine (100 mg/kg). Depth of anesthesia was assessed by monitoring the loss of the hind limb withdrawal reflex after pinch stimulation of the foot and the loss of eyelids reflex. Core temperature was maintained at 37 °C using a heating pad. Briefly, two groups of rats (*n* = 6 per group) received intranasally RBV agglomerates containing α-cyclodextrin (AM3) or micronized RBV as reference. In both cases, a 1 mg total dose of RBV was administered unilaterally to the left nostril with the animal lying horizontally on the right side.

RBV was administered inserting the tip of the DP-4 Dry Powder Insufflator™ device in the selected nostril of the rat. To reach the requested dose, 2–3 mg of the powder was manually filled into the device’s holding chamber immediately before the administration. The amount of RBV delivered was calculated based on the chamber’s weight difference before and after use.

Animals were sacrificed 20 min after administration of the powder, to collect plasma and the brain compartments of interest, i.e. olfactory bulb, cortex (anterior and posterior), basal ganglia, and hippocampus. Sample collection and processing were according to conditions reported by Colombo et al. (2011). After freezing in liquid nitrogen, samples were stored at −80 °C until analysis.

### Statistical analysis

The overall data obtained from the *in vivo* experiments were analyzed using an unpaired two-tailed Student’s *t*-test (Excel Software, Microsoft Corp., Redmond, WA). Statistical significance was accepted at *p* < .05.

## Results

The first part of the work attempted to formulate RBV as a powder for nasal insufflation focusing on the production of microparticles containing the antiviral drug along with the excipients selected. However, it was soon found that spray drying of RBV led to poor yields and unacceptable drug loss in the process due to drug accumulated as a sticky layer on the equipment glassware (data not shown). This was deemed a consequence of the substance’s known hygroscopic behavior (Lachenmann et al., 1990).

The alternative decision, then, was to use the pure micronized RBV crystals without processing and formulate them with spray-dried microparticles containing excipients able to favor the nasal administration and nose-to-brain absorption of the hydrophilic drug. RBV microcrystals then, together with microparticulate excipients with permeation enhancing properties, could be used to obtain a dosage form able to maintain the rapid dissolution characteristics of micronized crystals, improve the poor flowability of the micronized powder and exploit the functional properties of the excipients, which might facilitate the transport of the antiviral across the olfactory mucosa.

SEM images of RBV raw material and the spray-dried excipient powders have been included in Appendix provided as Supplemental File (Figure A1). The raw material presented a typical crystalline appearance with elongated, columnar or tabular shape characterized by a quite broad size distribution. In contrast, the spray-dried microparticles were spherical and homogenous micron-sized structures. In all three excipient particles, two populations of microparticles were identified, one population of larger particles with size around 10 µm and a second main population of smaller particles of few microns.

**Figure 1.**
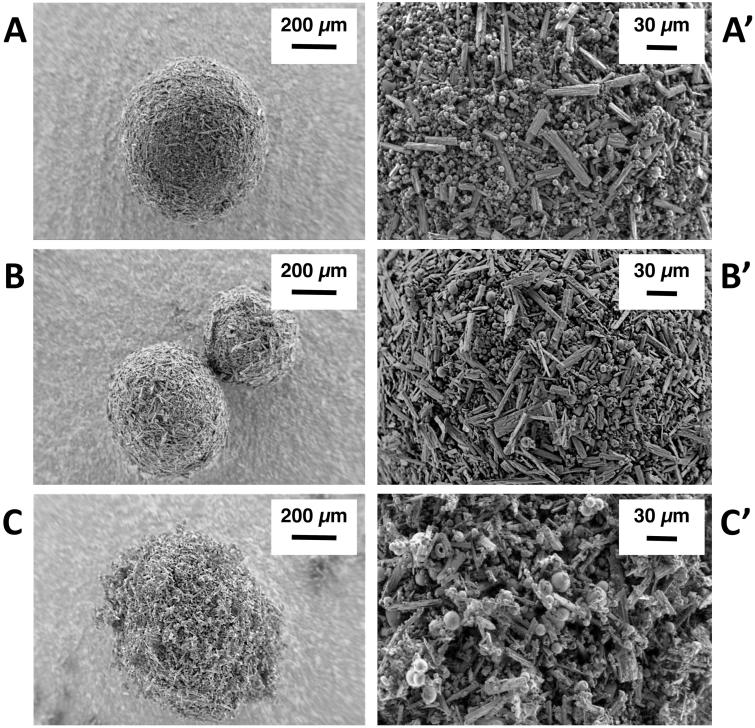
SEM images depicting the morphology of ribavirin-excipient agglomerates obtained with (A–A’) mannitol/lecithin microparticles (AM1); (B–B’) chitosan/lecithin microparticles (AM2), and (C–C’) α-cyclodextrin/lecithin microparticles (AM3) at 250× and 1200× magnification.

The excipients’ powder morphology was quite different. Mannitol–lecithin microparticles (M1) appeared as hollow shells with numerous evident cracks and fractures. Chitosan/lecithin microparticles appeared more regular than M1 and somehow denser. Finally, in the case of M3 powder, where α-cyclodextrin was employed along with lecithin, all particles were shriveled, suggesting the collapse of a dense shell over a hollow core during the drying process. Table A1 lists the production yield, particle size, and residual water content of the spray-dried microparticles and RBV raw material are provided also in the Appendix (Table A1 in Supplemental File).

**Table 1.** Yield of agglomeration (%), ribavirin content in the blends before agglomeration and in the final agglomerates produced (*n* = 3, average ± SD).

Blend 1:1	AgglomerationYield (%)	RBV Contentin Blend(% w/w)	RBV Content inAgglomerates(% w/w)
AM1	70.4 ± 3.2	53.8 ± 2.5	50.0 ± 4.2
AM2	80.2 ± 8.4	50.6 ± 0.5	53.7 ± 3.0
AM3	91.0 ± 5.2*	47.9 ± 1.2	47.9 ± 1.6

*Significantly different (*p* < .01) from agglomeration yield obtained for AM1.

The spray drying yields were around 50%. Particle sizing data confirmed that RBV was a micronized powder with a D_v,50_ volume diameter close to 10 µm and relatively broad size distribution (span value 2.36). In contrast, excipient microparticles presented a narrower size distribution (span value 2.00) with a D_v,50_ value between 6 and 7 µm. Microparticles containing chitosan and α-cyclodextrin had a relatively high residual water content as a result possibly of the lower inlet temperature in the spray drying process and of the hydrophilic nature of the polysaccharide excipients used in association with lecithin. On the contrary, M1 powder had a significantly lower residual water content reflecting the low hygroscopic behavior of its polyol component.

Agglomerates were prepared from a 1:1 mass ratio blend of the micronized RBV powder (µRBV) with each one of the excipient microparticles by vibration upon a sieve stack. Figure 1 shows the agglomerates obtained using the three excipient microparticles formulation. Agglomerates produced with mannitol/lecithin microparticles (AM1, Figure 1(A)) and chitosan/lecithin microparticles (AM2, Figure 1(B)) appear more spherical and densely packed. The agglomerates obtained with α-cyclodextrin/lecithin microparticles (AM3, Figure 1(C)) are bristly and less spherical. The observation of the agglomerates’ surface at higher magnification evidenced a homogenous distribution of the microparticles and micronized drug crystals.

Cumulative particle size distribution of the agglomerates obtained by optical microscopy, as well as the agglomeration yield, RBV content of the blend before agglomeration and RBV content of the agglomerates are included in the Appendix provided as Supplemental File.

The agglomerates obtained with mannitol/lecithin microparticles AM1 were those presenting a larger size distribution with 50% of the agglomerates between 350 and 400 µm. The same agglomerates showed also a lower percentage of particles below 300 µm. Since agglomerates were formed on a sieve with nominal aperture of 300 µm, it is expected that the presence of particles below this value correlates with the tendency of agglomerates to break or shed fragments. This suggests that AM1 agglomerates were likely stronger in terms of mechanical resistance, as smaller quantities of fragments or non-agglomerated powder were evidenced. The size distribution of agglomerates obtained with chitosan/lecithin (AM2) and α-cyclodextrin/lecithin microparticles (AM3) was superimposable, with half of the particles with a diameter around 350 µm.

The yield of agglomeration for the three mixtures was in the range between 70 and 90%, with the agglomeration efficiency of AM3 significantly higher than the one obtained for AM1 (*p* < .01). Furthermore, despite the differences observed in the macroscopic features of the agglomerates, their drug content was very close to the theoretical 50% according to the 1:1 ratio by weight of the initial mixture (see Table 1).

**Figure 2.**
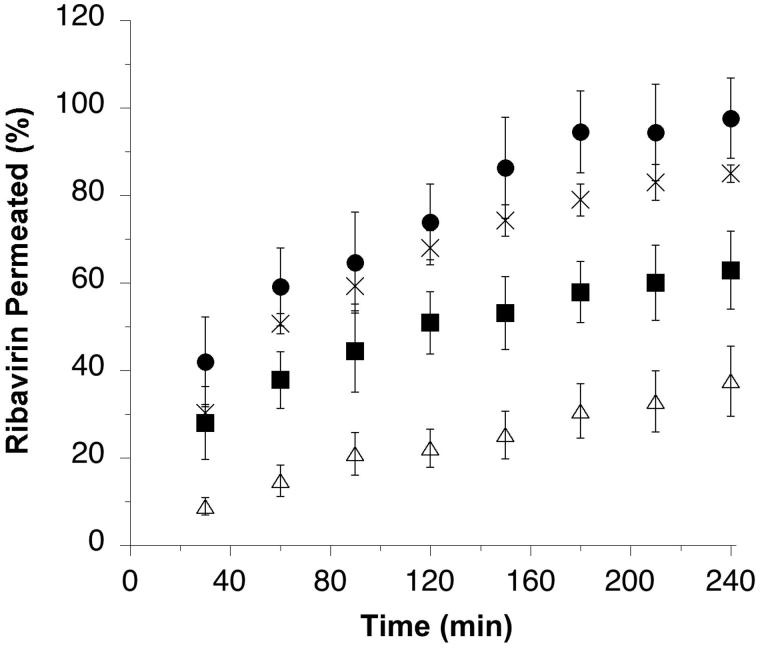
Ribavirin permeated (% of loaded dose) across rabbit nasal mucosal tissue from μRBV (×), and agglomerates obtained from micronized ribavirin crystals with mannitol/lecithin microparticles AM1 (▪), chitosan/lecithin microparticles AM2 (△) or α-cyclodextrin/lecithin microparticles AM3 (•) (*n* = 3, average ± SD).

In order to evaluate the biopharmaceutical characteristics of the agglomerates, the permeation of the antiviral drug *ex vivo* across rabbit nasal mucosa from the agglomerates was compared with that obtained from the micronized raw material as such (Figure 2).

The permeation of the antiviral drug was found to be relatively low in percentage from the agglomerates produced using chitosan/lecithin microparticles. Agglomerates produced using mannitol/lecithin microparticles showed a permeation similar to RBV microcrystals only until the first time point, then the total permeation after 4 hours was just below 60% of the loaded drug, thus significantly lower than the 80% obtained with the drug microcrystals. The micronized crystals and the agglomerates produced with α-cyclodextrin/lecithin microparticles showed instead a higher drug permeation leading to an almost complete diffusion across the biological barrier within the time of the experiment. In particular, the agglomerates containing α-cyclodextrin provided a total permeation after 4 h above 95%. The different results observed were explained considering that despite all the excipients selected for the agglomerates had the potential to enhance the permeation of a hydrophilic drug, their presence could have affected the drug dissolution. The slower the dissolution, the lower the drug concentration at the mucosal surface and slower the diffusion. Drug dissolution profiles were determined using the same Franz-type diffusion cells and a porous cellulose acetate membrane as barrier, showing faster dissolution for RBV microcrystals and for agglomerates obtained with α-cyclodextrin/lecithin microparticles (AM3), followed by those obtained with mannitol/lecithin microparticles (AM1) and chitosan/lecithin microparticles (AM2) (see Figure A3 in the Appendix in Supplemental File).

**Figure 3.**
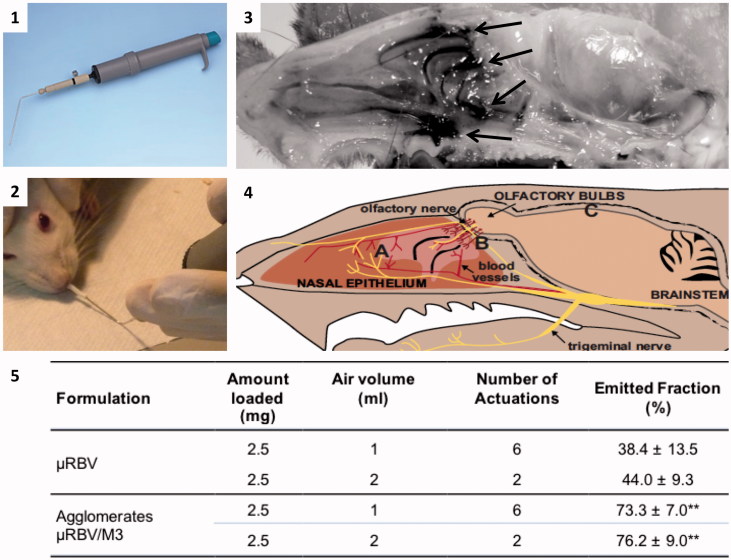
The panels are showing: (1) dry powder insufflator device; (2) tip insertion in the nose of an animal; (3) distribution in the nasal cavity of agglomerates containing a blue marker, arrows indicate regions where the staining is more evident; (4) sketch showing the relative positioning of the trigeminal nerve (A), olfactory nerves (B) and brain (C) in the animal head (reproduced with permission from Dhuria, 2010); (5) data related to *in vitro* powder delivery performance of the dry powder insufflator device (*n* = 4, mean ± SD); **significantly different from ?RBV emitted powder, *p* < .01.

In light of the results of the *ex vivo* permeation experiment, the agglomerates containing the micronized RBV crystals with α-cyclodextrin/lecithin microparticles were selected for the *in vivo* study of RBV brain distribution after nasal administration.

For the administration of the powders into the animal’s nose, a dry powder insufflator available for the pulmonary administration of powders to small animals was used. A preliminary study *ex vivo* was carried out to determine the device’s optimal positioning and evaluating powder deposition in the rat nasal cavity. Figure 3 shows the device (Figure 3.1), the positioning of its tip within the nostril of the animal (Figure 3.2) and the distribution of the agglomerate powder after insufflation (Figure 3.3 and 3.4). The agglomerate powder loaded with a blue dye (black areas in Figure 3.3) is marking the deeper section of the animal nasal cavity where the olfactory and trigeminal innervation is present (Dhuria, 2010). The preliminary experiment carried out here was necessary to assess the distribution of the powder in the nasal cavity and rule out the formation of a clog or lumps at the site of insufflation.

**Figure 4.**
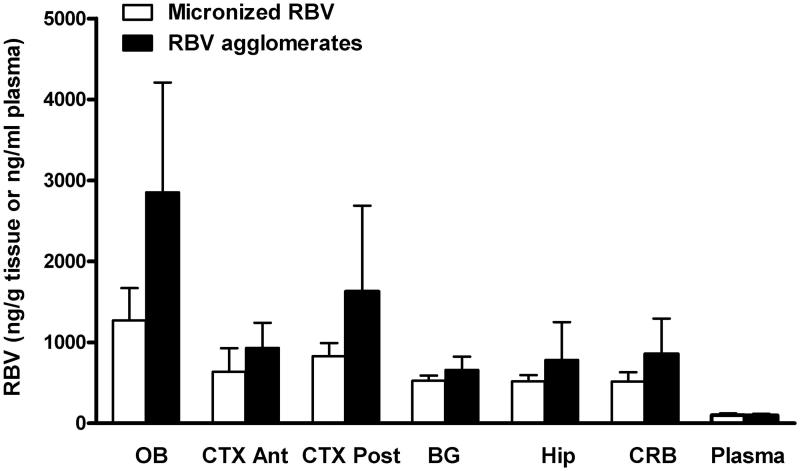
Ribavirin distribution in plasma and different brain compartments (OB: olfactory bulb; BG: basal ganglia; Hip: hippocampus; CTX Ant: anterior cortex; CTX Post: posterior cortex; CRB: cerebellum) after the nasal administration of micronized ribavirin crystals (white bars) or agglomerates obtained with micronized ribavirin and α-cyclodextrin/lecithin microparticles (AM3) (black bars) (*n* = 6, average ± SEM).

The *in vitro* performance of the device when loaded with the agglomerates and with the micronized RBV powder in terms of air volume and number of actuations for insufflation, and percentage emitted powder is presented in Figure 3.5. As expected, µRBV was more difficult to aerosolize for nasal insufflation. In fact, in the case of the micronized crystals the device delivered less than 50% of the loaded amount of powder either with high or low air volume setting. In contrast, agglomerates delivered a significantly higher amount of powder in both conditions and only two actuations were sufficient to deliver more than 75% of the loaded amount of powder when the device was operated with the higher volume of air. The observed results can be related to the improved flowability of the agglomerated powder compared to the micronized RBV, as shown by the calculated Carr indexes. In fact, CI values were 10 ± 3 for AM3 agglomerates and 28 ± 1 for micronized RBV, corresponding to excellent and poor flow characteristics according to the European Pharmacopoeia scale of flowability.

**Figure 5.**
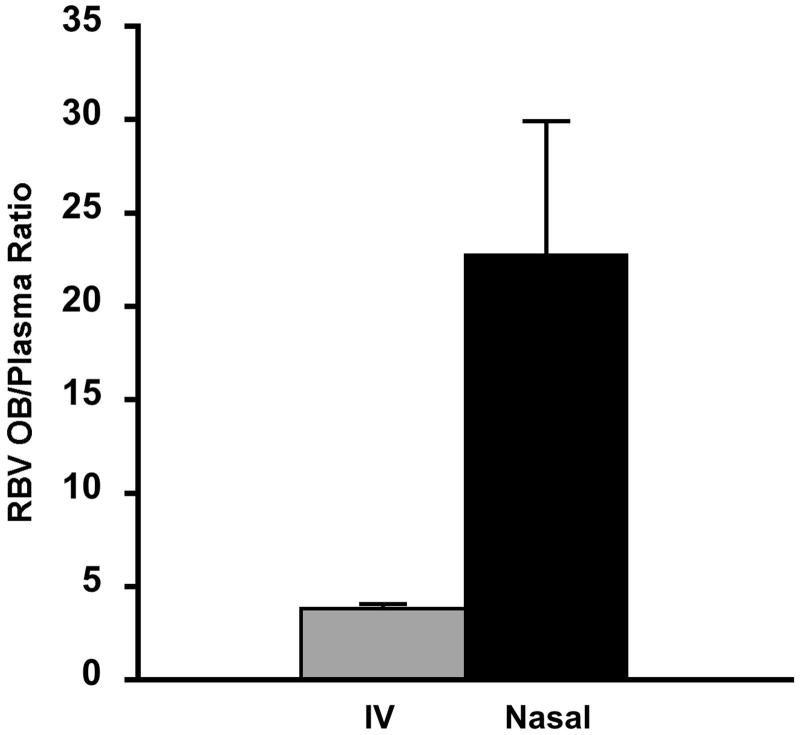
Olfactory bulb to plasma ratio (OB/plasma ratio) after nasal administration of the same dose of ribavirin (1 mg) by intravenous injection (grey bar) or nasal administration of ribavirin agglomerates with α-cyclodextrin excipient microparticles (black bar) (*n* = 6, average ± SEM). Data for the intravenous administration are according to Colombo et al., 2011.

Based on the device performance *in vitro*, for the *in vivo* experiments it was decided to use the device in the following conditions: 2 ml air volume and not more than two insufflations, in order to maximize the powder delivered without stressing the animals. The exact amount delivered to each animal was determined by accurately weighing the device before and after the insufflation.

After the nasal administration of the formulations *in vivo*, animals were sacrificed 20 min post-treatment and the amount of RBV accumulated in various brain regions quantified and normalized for the RBV dose actually administered. RBV was quantified in the olfactory bulb and plasma, as well as in basal ganglia, hippocampus, anterior and posterior cortex and cerebellum. As shown in Figure 4, the highest concentrations of RBV were found in the olfactory bulb followed by the posterior and anterior cortex and the other brain regions. Plasma concentration was comparably low. Agglomerates outperformed the micronized RBV powder producing relatively elevated levels of antiviral in all the brain regions analyzed. The differences between the two groups, however, were not significant.

In order to evaluate the actual contribution of direct nose-to-brain transport of RBV to the levels measured, the concentrations in the olfactory bulb obtained here by nasal administration of the agglomerates were compared with those obtained previously with the intravenous administration of the same RBV dose per kg of body weight (Colombo et al., 2011). This allowed for the calculation of the olfactory bulb to plasma ratio (OB/plasma ratio) for both administration routes (Figure 5). The OB/plasma ratio values supported the direct nose-to-brain transport of the antiviral drug after nasal administration of the RBV powder agglomerates. The OB/plasma ratio value was significantly different (*p* < .05) and nearly 6-fold as high in the case of the nasal administration of the agglomerates as for the intravenous administration.

## Discussion

Despite the field of nasal delivery is dominated by liquid sprays, the use of dry powder formulations appears appealing for innovative products, in particular those designed for a nose-to-brain delivery (Tiozzo Fasiolo et al., 2018). In fact, dry powder formulations are providing several advantages over liquids such as chemical stability, absence of preservatives, higher unit dosages per administration, longer residence time and contact with the nasal mucosa and eventually improved bioavailability (Pozzoli et al., 2016). However, not always the formulation of powders to be delivered to the nose allows to combine together some of the most desirable features. Ideally, particles should be sufficiently large to obtain a good deposition in the nasal cavity, but at the same time display a high surface area for fast dissolution. Mucoadhesive or permeation enhancer excipients might be included as well. One popular strategy is to use particle engineering to achieve these goals. Spray drying is a technique that has elicited a lot of interest for pharmaceutical particle engineering because of its versatility in term of obtainable particle morphology, size, and density and possibility to combine multiple components in the particle processing (Vehring, 2008). Spray-dried particles have been proposed both for the delivery of drugs in form of dry powders both to the upper (Russo et al., 2006; Dalpiaz et al., 2008; Chen et al., 2013) and the lower respiratory tract (Xu et al., 2012; Chan et al., 2013; Belotti et al., 2015).

In the case of RBV, however, the impossibility of obtaining spray-dried microparticles containing RBV at the desired high drug content was evidenced, being the result of the hygroscopic behavior of the drug (Lachenmann et al., 1990). Interestingly, it has been reported that handling of RBV poses considerable challenges as the raw material is subjected to pharmaceutically relevant process-induced solid state changes (Vasa & Wildfong, 2017). This has led to a different exploitation of the particle engineering approach, here applied to the manufacturing of functional excipients microparticles followed by the production of organized mixtures of such microparticles with micronized crystals of the antiviral drug in the form of spherical agglomerates.

In fact, due to the particle size distribution of the micronized RBV powder and of the excipient microparticles, simple powder blends of these two components are not suitable for nasal delivery. In fact, both the technological (poor flow and high cohesiveness) and biopharmaceutical properties (limited deposition in the nasal cavity and inhalation into deeper airway sections for particles below 10 µm) of micronized powders make them not suitable for nasal administration. However, the agglomeration of micron-sized powders, like the present primary particles, is an elegant solution that allows us to change the size distribution of the powder. This improves flow and handling, while it retains other favorable properties of the original powders, such as original solid state, high surface area, and rapid dissolution. In addition, the large agglomerates or their fragments can be deposited in the nasal cavity by impaction and reduce particle inhalation into the lungs (Russo et al., 2006; Balducci et al., 2013b).

In terms of particle size distribution and shape, the spray-dried excipients microparticles were similar to those obtained previously by ours and other groups. The differences in particle structure (dense, hollow, shriveled) have been attributed to the slightly different drying conditions applied, but mostly to the behavior of the components during the drying process, i.e. their preferential absorption on the particle surface or diffusion away from the surface, as already described and exploited to obtain low-density microparticles (Tsapis et al., 2002; Belotti et al., 2014). Here, mannitol, a small molecule with higher diffusion coefficient, led to full spheres, chitosan, a large polymer, to shriveled particles, very similar to those obtained for protein-based spray dried particles (Balducci et al., 2014) and α-cyclodextrin, an oligosaccharide having surface active properties, led to the production of hollow microparticles. Those shape differences could be attributed to the effect of molecular weight and solubility in the liquid feed of the three selected excipients.

However, despite the different particle structures, the agglomeration properties of the microparticles were not substantially different among the three compositions and this was attributed to the lecithin presence at the same concentration in all cases. All compositions, in fact, produced agglomerates with satisfactory process yields. It is possible that the excipient microparticles residual water content played also a role in favoring the agglomeration process, since M2 and M3 spray-dried particles producing agglomerates with micronized RBV crystals with yields above 80%, showed a significantly higher residual humidity compared to M1 microparticles. The actual RBV content in the agglomerates reflected the drug content determined for the blend before agglomeration, indicating a homogenous incorporation of the drug in the agglomerates with no drug loss or enrichment caused by the agglomeration process.

However, differences became evident with respect to RBV permeation *ex vivo* across rabbit nasal mucosa. RBV is a highly hydrophilic and water-soluble molecule and consequently its permeation across the biological barrier is expected to occur relatively slowly (Costantino et al., 2007; Illum, 2012). It was confirmed that the permeation occurred at great extent when the drug was in the form of micronized crystals, due to the intimate contact with the lining fluid of the mucosa, the rapid dissolution and the concentration gradient achieved, that was the highest possible (Buttini et al., 2012; Tiozzo Fasiolo et al., 2018). Quite surprisingly, agglomerates containing chitosan/lecithin microparticles were those that led to a significantly lower permeated amount compared to the micronized RBV. Chitosan has been indicated as the excipient of choice for nasal delivery because it acts as an absorption promoter with a double mechanism of action: it acts as a mucoadhesive agent due to electrostatic interactions with mucus components, and as a permeability enhancer, transiently loosening tight junctions of nasal epithelial cells (Casettari et al., 2012). On the one hand, many authors reported improvements in drug mucosal permeation as a consequence of the inclusion of chitosan in solution (Illum et al., 1996), powder blends (Illum et al., 2002; Chen et al., 2010), inserts (Luppi et al., 2010), nanoparticles (Wang et al., 2009; Barbieri et al., 2015), and microparticles (Gavini et al., 2013). On the other hand, some other reports indicate that those permeation enhancing properties were diminished or not evidenced in the case of nanoparticulate formulations (Dyer et al., 2002; Illum, 2007) or when the microparticulate formulation showed controlled release of the drug (Dalpiaz et al., 2008). In the present case, the observed lack of permeation enhancement with RBV could be related to similar reasons. In fact, chitosan provides a more remarkable effect on the permeability of the mucosal tissue when it is in form of molecular dispersion. It can be hypothesized that the chitosan in the M2 microparticle composition did not dissolve rapidly when deposited on the mucosal surface. Moreover, this slow dissolution may have passed through the hydration and formation of a gel that eventually slowed down the dissolution of the RBV as well, leading to a gradual release and relatively slow diffusion. Indeed, Tanaka et al. (2017) recently attributed the reduced nasal absorption of warfarin, a highly soluble and highly permeable drug, from formulations containing different types of hydroxypropylcellulose (HPC) to the delayed dissolution and slow diffusion of the drug in the viscous fluid created by the polymer.

Mannitol is a safe non-ionic highly water soluble pharmaceutical excipient that has been recently used as hyperosmotic agent in the treatment of chronic obstructive pulmonary diseases, such as cystic fibrosis and bronchiectasis (Hirsh, 2002; Young et al., 2014). The same hyperosmotic effect is considered to improve paracellular transport of hydrophilic drugs in epithelia and endothelia, suggesting a mannitol-induced tight junction opening that has been used also to induce disruption of the BBB (Deli, 2009). The permeation of RBV across rabbit nasal mucosa from agglomerates containing mannitol and lecithin also failed to outperform the micronized RBV crystals. In this case, the mannitol present in microparticles is expected to have dissolved rapidly, causing a rapid water uptake from the mucosal surface. However, this high amount of water molecules interacting with mannitol could have slowed down the dissolution of RBV crystals, in a manner comparable with the well-known poor solvent properties of syrups.

Cyclodextrins have been used for nasal drug delivery as solubilizers, but also as penetration enhancers (Colombo et al., 2016). In particular, the latter function has been evidenced for α-cyclodextrin (Merkus et al., 1999). Quite interestingly the complexation of RBV with α-cyclodextrin via the formation of an external intermolecular complex was demonstrated (Grancher et al., 2005) and its antiviral activity in the brain after intraperitoneal administration was evidenced previously (Jeulin et al., 2008, 2009). As a consequence, α-cyclodextrin was attractive for the preparation of spray-dried excipient microparticles for nasal delivery. Indeed, as far as *ex vivo* permeation was concerned, the agglomerates containing α-cyclodextrin and lecithin outperformed the others and even provided a higher permeation than the micronized powder. This could be related to a rapid RBV dissolution, to the contribution to the concentration gradient of cyclodextrin complexed drug and to a direct effect on the nasal mucosal tissue.

In addition, agglomerates showed excellent flow properties, as demonstrated by the calculated Compressibility Index, and in preliminary experiments of insufflation with a device suitable for animal nasal administration performed significantly better than the micronized RBV and provided an extensive distribution in the animal nasal cavity.

The study in animals showed that agglomerates containing α-cyclodextrin and lecithin determined higher brain RBV concentrations compared with the micronized powder administered as such, even if the values were not significantly different (possibly as a consequence of the short time set for the *in vivo* experiment and the single time point acquired). Interestingly, the high RBV levels in both the posterior cortex and cerebellum suggest an involvement of trigeminal nerve pathway in the absorption of the antiviral drug (Dhuria, 2010). However, it may be argued that the vascular pathway (RBV absorption into the blood and BBB crossing) contributed to the brain delivery, based on the reports on the RBV complex with α-cyclodextrin enabling crossing the BBB (Jeulin et al., 2009). For this reason, RBV levels previously measured after intravenous administration of the same drug dose per kg of body weight were directly compared with those obtained here with the nasal agglomerates. The concentrations in the olfactory bulb were found not to be significantly different. Considering that nasal bioavailability of hydrophilic drugs is estimated lower than 10% (Casettari & Illum, 2014) and that in most of studies the nasal dose administered is 10-fold the injected one, the result is quite promising in view of the nasal administration of this antiviral drug, especially considering the possibility of repeating the administration.

Very interestingly, when the olfactory bulb-to-plasma concentration ratios were calculated as a measure of the nose-to-brain targeting efficiency (Serralheiro et al., 2015), the brain targeting effect of the nasal administration was evident together with lower plasma concentrations, providing the substantial possibility to reduce systemic side effects.

## Conclusions

RBV was successfully formulated for nose-to-brain administration in the form of agglomerates with spray-dried excipient microparticles suitable for efficient nasal deposition and able to provide mucoadhesion and permeation enhancing properties. Despite being quite similar in their physico-chemical properties, the agglomerates produced showed remarkable differences with respect to permeation of the antiviral drug across rabbit nasal mucosa. The differences were attributed to the relative effect of the excipients in the microparticle composition on the dissolution behavior of the drug. The agglomerates containing α-cyclodextrin and lecithin microparticles enhanced the permeation compared to micronized drug crystals. Results *in vivo* confirmed the potential of the formulation for nose-to-brain delivery of the antiviral drug with high brain targeting also in comparison with a conventional intravenous administration.

The technological approach presented, i.e. the agglomeration of excipient microparticles combined with the drug raw material, has the potential to be a platform for the nose-to-brain drug delivery of hydrophilic small molecules, but also of peptides and proteins. In fact, it does not require the processing of the pharmacologically active substance, maximizing its stability, and at the same time combines excipients useful for biopharmaceutical events such as dissolution, mucoadhesion and enhancement of the permeation through the nasal epithelium, ultimately affecting drug bioavailability.

## Supplementary Material

IDRD-Sonvico_et_al_Supplemental_Content.docx
