# Supplementary material for: In vivo nose-to-brain delivery of the hydrophilic antiviral ribavirin by microparticle agglomerates
Source: Drug Deliv. 2018 Jan 30;25(1):376–87. doi: 10.1080/10717544.2018.1428242 (PMC6058489; doi:10.1080/10717544.2018.1428242)
Supplement: IDRD-Sonvico_et_al_Supplemental_Content.docx [file IDRD_A_1428242_SM7572.docx]

***In vivo* brain delivery of a hydrophilic antiviral drug by nasal permeation enhancing microparticle agglomerates**

**APPENDIX**

Table A1. Yield of spray drying process (%), particle size distribution (µm) and water content of ribavirin raw material and excipient spray-dried microparticles (n=3, average ± SD)

| **Powder** | **Yield (%)** | ***D_v_,_10_ (µm)*** | ***D_v_,_50_ (µm)*** | ***D_v_,_90_ (µm)*** | **Water Content (%)** |
| --- | --- | --- | --- | --- | --- |
| **RBV** | n/a | 3.30 ± 0.64 | 11.63 ± 1.83 | 30.81 ± 3.62 | 0.58 ± 0.09 |
| **M1** | 43.6 ± 11.2 | 2.44 ± 0.15 | 7.38 ± 0.98 | 16.64 ± 3.30 | 1.45 ± 0.10 |
| **M2** | 48.5 ± 14.2 | 2.30 ± 0.35 | 7.14 ± 2.48 | 17.21 ± 3.14 | 5.98 ± 0.12 |
| **M3** | 59.6 ± 5.3 | 2.62 ± 0.49 | 6.10 ± 0.21 | 14.79 ± 4.8 | 6.75 ± 0.17 |


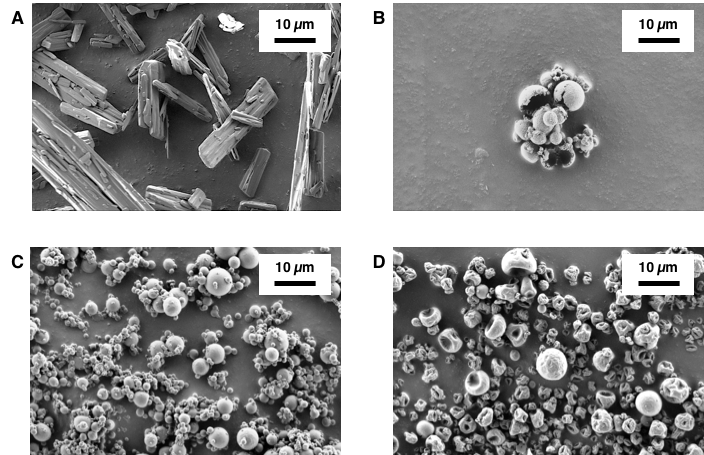


Figure A1. SEM images (5000X magnification) depicting the morphology of: A) micronized ribavirin raw material; B) lecithin and mannitol spray-dried powder (M1), C) lecithin and chitosan spray-dried powder (M2) and D) lecithin and α-cyclodextrin spray-dried powder (M3).


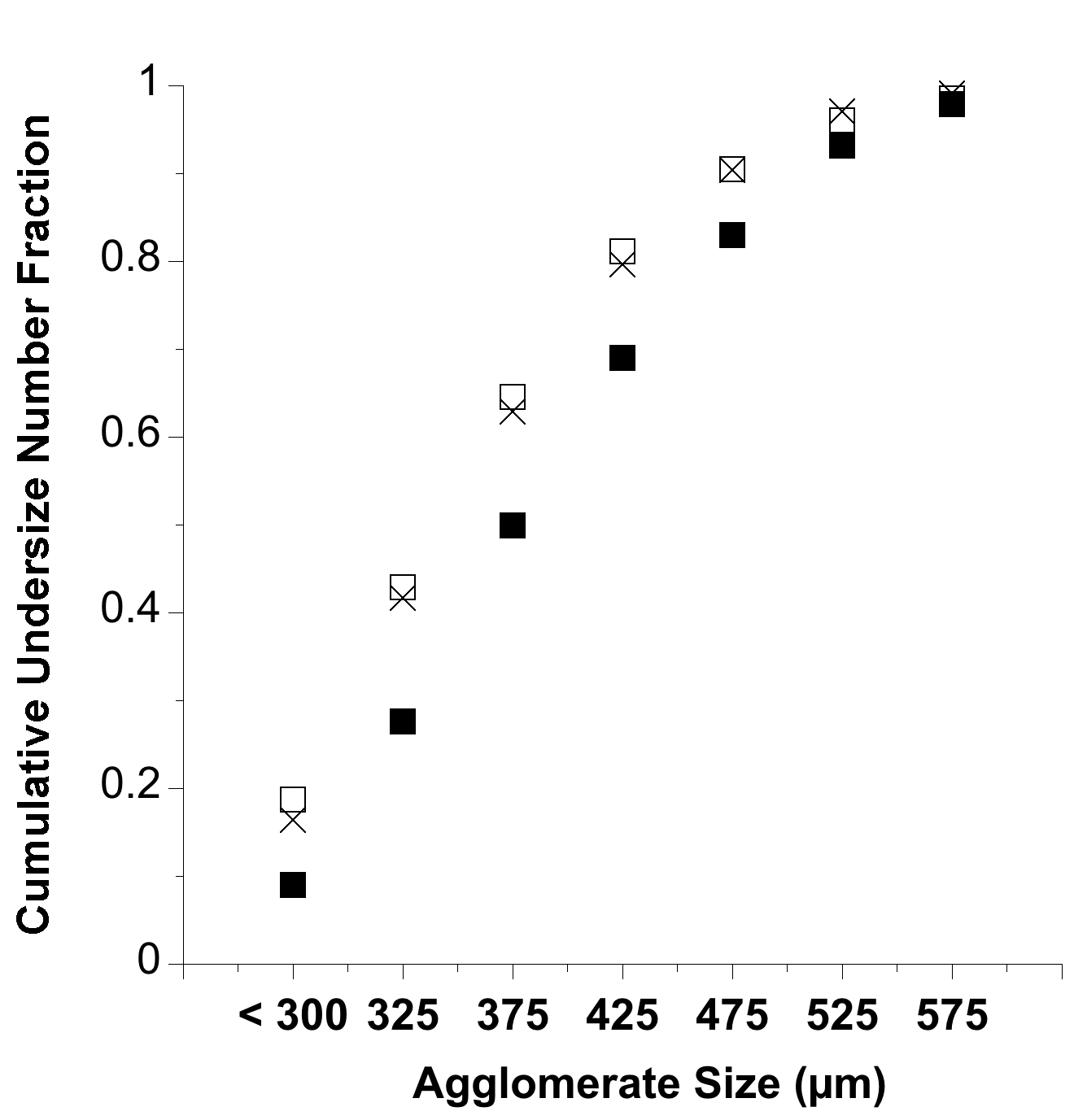


Figure A2. Cumulative undersize distribution by number of agglomerates obtained using micronized ribavirin and mannitol/lecithin microparticles M1 (■), chitosan/lecithin microparticles M2 (□) and α-cyclodextrin/lecithin microparticles M3 (🞨).


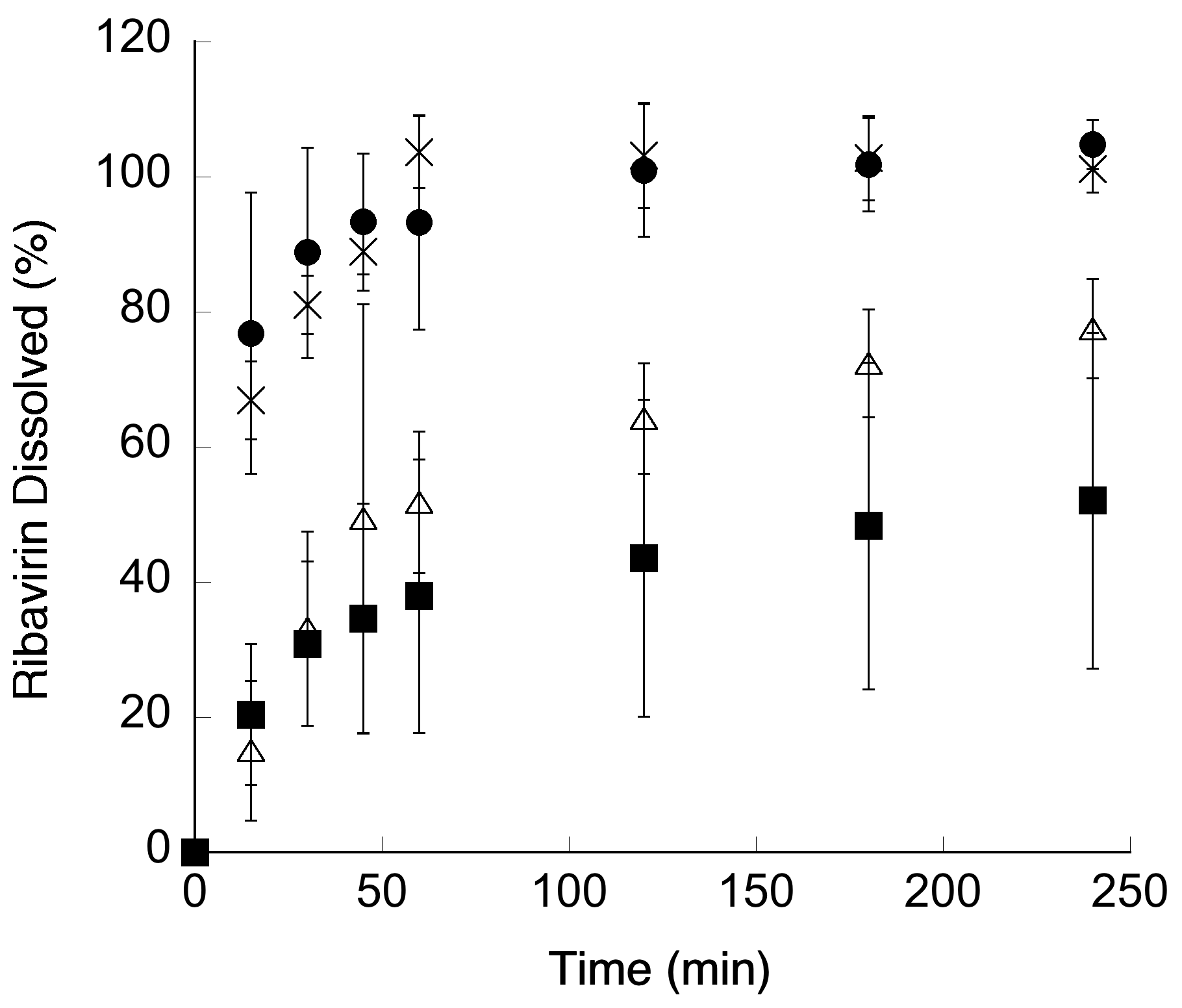


Figure A3. Ribavirin dissolved (% of the loaded dose) across porous cellulose acetate membrane (0.45 µm) from µRBV (**🞨**), and agglomerates obtained from micronized ribavirin crystals with mannitol/lecithin microparticles AM1 (■), chitosan/lecithin microparticles AM2 (**△**) or α-cyclodextrin/lecithin microparticles AM3 (●) (n=3, average ± SD).
